# Host factors determine differential disease progression after infection with *nef*-deleted simian immunodeficiency virus

**DOI:** 10.1099/vir.0.066563-0

**Published:** 2014-10

**Authors:** Sieghart Sopper, Kerstin Mätz-Rensing, Thorsten Mühl, Jonathan Heeney, Christiane Stahl-Hennig, Ulrike Sauermann

**Affiliations:** 1 Tumor Immunology Lab, Hematology and Oncology, Medical University Innsbruck and Tyrolean Cancer Research Institute, Innsbruck, Austria; 2 Pathology Unit, German Primate Center, Leibniz Institute for Primate Research, Goettingen, Germany; 3 Unit of Infection Models, German Primate Center, Leibniz Institute for Primate Research, Kellnerweg 4, 37077 Goettingen, Germany; 4 Department of Veterinary Medicine, The University of Cambridge, Cambridge, UK

## Abstract

Infection of macaques with live attenuated simian immunodeficiency virus (SIV) usually results in long-lasting efficient protection against infection with pathogenic immunodeficiency viruses. However, attenuation by deletion of regulatory genes such as *nef* is not complete, leading to a high viral load and fatal disease in some animals. To characterize immunological parameters and polymorphic host factors, we studied 17 rhesus macaques infected with attenuated SIVmac239ΔNU. Eight animals were able to control viral replication, whereas the remaining animals (non-controllers) displayed variable set-point viral loads. Peak viral load at 2 weeks post-infection (p.i.) correlated significantly with set-point viral load (*P*<0.0001). CD4^+^ T-cell frequencies differed significantly soon after infection between controllers and non-controllers. Abnormal B-cell activation previously ascribed to Nef function could already be observed in non-controllers 8 weeks after infection despite the absence of Nef. Two non-controllers developed an AIDS-like disease within 102 weeks p.i. Virus from these animals transmitted to naïve animals replicated at low levels and the recipients did not develop immunodeficiency. This suggested that host factors determined differential viral load and subsequent disease course. Known Mhc class I alleles associated with disease progression in SIV WT infection only marginally influenced the viral load in Δ*nef*-infected animals. Protection from SIVmac251 was associated with homozygosity for MHC class II in conjunction with a *TLR7* polymorphism and showed a trend with initial viral replication. We speculated that host factors whose effects were usually masked by Nef were responsible for the different disease courses in individual animals upon infection with *nef*-deleted viruses.

## Introduction

Infection or immunization with live attenuated human immunodeficiency virus (HIV) or simian immunodeficiency virus (SIV) protects efficiently against symptoms of AIDS or infection with pathogenic immunodeficiency viruses (Daniel *et al.*, 1992; Gorry *et al.*, 2007; Koff *et al.*, 2006; Whitney & Ruprecht, 2004). The commonly used live attenuated viruses for experimental immunization of macaques harbour deletions in the viral *nef* gene. Vaccination with live attenuated viruses is not only very potent in terms of protection against challenge with pathogenic virus, but also with respect to its durability. As early as 3 weeks and as late as 2 years after immunization, juvenile and adult macaques can be protected against superinfection with pathogenic SIV (Sharpe *et al.*, 2004; Stebbings *et al.*, 2004). The immune mechanisms conferring protection have proven to be difficult to identify. Several reports have shown that protection can be obtained in the presence of low or even absent cellular or humoral immune responses (Mansfield *et al.*, 2008; Nilsson *et al.*, 1998; Stahl-Hennig *et al.*, 2007; Stebbings *et al.*, 2005).

The idea to immunize humans against HIV with live attenuated viruses has been dismissed because many if not all deletion constructs can ultimately cause disease in infected infants and, after prolonged observation, also in adult monkeys (Baba *et al.*, 1999; Chakrabarti *et al.*, 2003; Connor *et al.*, 1998; Hofmann-Lehmann *et al.*, 2003; Sawai *et al.*, 2000). Progression to immunodeficiency has also been observed in humans infected by blood transfusion from a HIV-1-infected donor carrying a deletion in *nef* (Gorry *et al.*, 2007). In addition, superinfection with pathogenic SIV can lead to recombination events and thus to the generation of novel pathogenic viruses (Gundlach *et al.*, 2000; Reynolds *et al.*, 2008).

Viral load is generally lower in monkeys infected with SIVmacΔ*nef* compared with WT SIV-infected macaques, but large individual differences can be observed, especially in rhesus macaques of Indian origin. Many factors may contribute to these differences and consequently to the virulence of attenuated SIV. The unique, not fully developed neonatal immune system is clearly a risk factor. Furthermore, partial refilling of the *nef* deletion leading to the production of a truncated viral Nef protein as well as a series of other alterations in the viral genome may increase the virulence of the virus (Alexander *et al.*, 2003; Sawai *et al.*, 2000; Whatmore *et al.*, 1995). In addition, host factors may determine differential viral replication and disease manifestation. Among the host factors influencing disease progression in immunodeficiency virus-infected humans and monkeys, polymorphic genes encoded in the MHC region represent the most important factors (Fellay *et al.*, 2009; Siddiqui *et al.*, 2009). In addition, polymorphisms within the Toll-like receptor 7 (*TLR7*) have been reported to modulate disease progression in HIV-1-infected humans and SIV-infected macaques (Oh *et al.*, 2009; Siddiqui *et al.*, 2011).

As the factors responsible for differences in disease progression after infection with SIVmacΔ*nef* have not yet been determined fully, the aim of this study was to characterize immunological parameters as well as host factors that contributed to differential disease outcome after experimental infection of rhesus monkeys with the SIVmac239ΔNU strain that carries a 513 bp deletion in *nef* (Gundlach *et al.*, 1997). We performed transmission experiments to investigate whether host factors or an enhanced virulence of the virus were responsible for high viral replication in some macaques. The results showed that host factors were important for viral containment. However, they may be different from those influencing pathogenic SIV infection.

## Results

### Variation of viral load and disease progression in rhesus monkeys infected with *nef*-deleted SIVmac

Seventeen rhesus macaques (*Macaca mulatta*) of Indian origin were infected with SIVmac239ΔNU (Gundlach *et al.*, 1997) either intravenously or via the tonsils. The infection route did not significantly influence viral RNA copies in plasma, at peak viraemia 2 weeks post-infection (p.i.) or at viral set point (24 weeks p.i.) (*P* = 0.174 and *P* = 0.81, respectively; Mann–Whitney test). Eleven macaques were observed for at least 64 weeks p.i. The other six macaques were challenged with SIVmac251 at 26 weeks p.i. (Tables 1 and S1, available in the online Supplementary Material) (Tenner-Racz *et al.*, 2004).

**Table 1.** Animal ID, route of infection, viral load at set point, observation time, and MHC and *TLR7* genotype

Animal ID	Route of infection*	Plasma viral RNA copies ml^−1^ at 24/26 weeks p.i.	Survival (weeks p.i.)	Disease course predicted by MHC class I genotype	Homozygous for MHC class II *DQB1-DRB* haplotype	*TLR7* genotype associated with slow disease progression†
**Controllers**						
1937	I.v.	Negative	>64‡	Rapid	Yes	Yes
1939	Tonsils	Negative	>26‡	Slow	No	Yes
1964	I.v.	Negative	>26‡	Rapid	Yes	Yes
1982	I.v.	Negative	>26‡	Slow	Yes	Yes
8638	Tonsils	Negative	>62	Unknown	No	No
8785	I.v.	Negative	>64‡	Unknown	Yes	Yes
8790	I.v.	220, 32 weeks p.i. Negative	>64‡	Moderate	Yes	No
9026	I.v.	Negative	>64‡	Moderate	No	Yes
**Non-controllers**						
1891	I.v.	29 000	102	Unknown	Yes	Yes
1948	I.v.	54 000	100	Unknown	No	Heterozygous
1961	Tonsils	7900	84	Unknown	No	Yes
1968	Tonsils	1000	>26‡	Unknown	Yes	No
1969	i.v.	3200	>26‡	Rapid	No	No
8637	Tonsils	680	>62‡	Unknown	No	Yes
8768	I.v.	560	180	Unknown	Yes	Yes
9037	Tonsils	1400	>26‡	Unknown	No	Yes
9038	I.v.	2500	185	Unknown	No	No
**Transfused**						
1891T	Material from Mm1891; i.v.	1200	58	Unknown	No	Yes
1948T	Material from Mm1948; i.v.	1600	61	Moderate	No	No

* I.v., Intravenous; tonsils, atraumatic via tonsils.

† *TLR7* genotype associated with slow disease progression: c.−17C, c.13G (Siddiqui *et al.*, 2011).

‡ Animals were challenged with SIVmac251 at the indicated time point and excluded from further analysis.

**Fig. 1.**
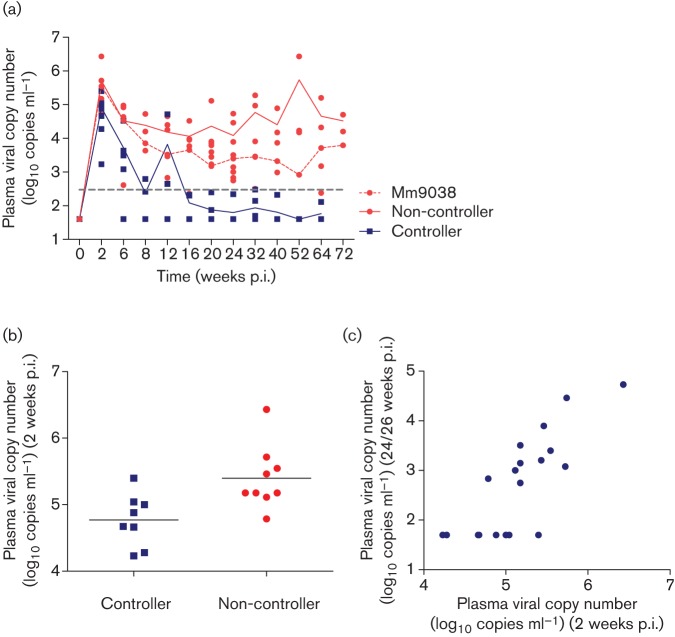
Plasma viral RNA copy numbers (log_10_) in 17 SIVmac239ΔNU-infected macaques over time. (a) Geometric means of controllers (blue) and non-controllers (red) are indicated by solid lines. Data of individual animals are shown by dots. Plasma RNA copy number of the viraemic controller Mm9038 is shown by a dashed line. Set-point plasma viral load (300 RNA copies ml^−1^) at 24 weeks p.i. used to distinguish between controllers and non-controllers is indicated by a grey dashed line. (b) Plasma viral RNA copy number 2 weeks p.i. distinguishes controllers from non-controllers (*P* = 0.0055, Mann–Whitney test). (c) RNA copy number at 2 w.p.i. correlates with set-point viral load (*P*<0.0001, *r* = 0.82, Spearman rank correlation).

In all monkeys, the initial peak viraemia was reduced by one to four orders of magnitude within the first 24 weeks p.i., coinciding with the onset of an immune response (Fig. 1a, Table S1) that resulted in a wide variation of set-point viraemia. Based on the viral load at 24/26 weeks p.i., we stratified the animals in two groups: the controllers, displaying a plasma viral load <300 RNA copies ml^−1^, and the non-controllers, having a plasma viral load >300 RNA copies ml^−1^ (Tables 1 and S1). The controllers (*n* = 8) were inoculated with pathogenic SIVmac251 at 26 or 64 weeks p.i. As expected, they maintained, at least initially, their controller phenotype, indicating that they had developed a vaccine-induced protective anti-SIV immune response (Stahl-Hennig *et al.*, 2007; Tenner-Racz *et al.*, 2004). The non-controllers (*n* = 9) replicated the virus at varying levels at the viral set point (20–26 weeks p.i.). With the exception of three animals, they were observed for at least 62 weeks p.i. Three macaques (Mm1968, Mm1969 and Mm9037) were challenged with WT SIVmac251 at 26 weeks p.i. They were included as their early viral replication kinetics clearly distinguished them from the controllers (Table 1, Fig. 1). Mm1968 and Mm1969 replicated SIVmac251 at time of death (Freissmuth *et al.*, 2010). Discrimination between SIVmac239ΔNU and SIVmac251 was not performed for Mm9037.

Plasma viral RNA copies measured at 2 weeks p.i. before onset of adaptive immune responses already distinguished between controllers and non-controllers (*P* = 0.0055, Mann–Whitney test, Fig. 1b), and correlated significantly with set-point viral load (*P*<0.0001, Spearman rank correlation, Fig. 1c).

In contrast to the controllers, non-controllers showed signs of immunological alterations at time of death after a prolonged observation period (Table 2). Two macaques (Mm1891 and Mm1948) that replicated the virus at high levels with a plasma viral load >10^4^ RNA copies ml^−1^ plasma at set point had to be euthanized with symptoms of AIDS at 100 and 102 weeks p.i., respectively (Tables 1 and 2). They both developed severe thrombocytopenia by 88 weeks p.i. In addition, Mm1948 presented with severe anaemia and opportunistic infections in lung and gut. Furthermore, necropsy revealed that both animals had a non-bacterial thrombotic endocarditis often detected in HIV-1-infected patients (Kaul *et al.*, 1991), SIV-associated arteriopathy of the small lung vessels and a severe generalized hyperplasia of the lymphatic organs. Mm1961, which also replicated SIVmac239ΔNU to high levels, was euthanized at 84 weeks p.i. with signs of generalized lymphatic hyperplasia in various organs, including gut-associated lymphoid tissues. Pneumonia was an additional finding in this animal.

**Table 2.** Findings at time of necropsy of SIVmacΔNU-infected monkeys

Animal ID	Parasitological results	Microbiological results	Pathohistological findings*
1891	–	–	Non-bacterial thrombotic endocarditis, severe SIV-associated arteriopathy of small lung vessels, severe generalized hyperplasia of LNs, spleen, GALT and BALT
1948	Gut: massive cryptosporidia, *Entamoeba* sp., *Giardia* sp.	Lung: *Streptococcus* sp.; gut: *Klebsiella* sp.	Non-bacterial thrombotic endocarditis, severe SIV-associated arteriopathy of small lung vessels, severe generalized hyperplasia of LNs and spleen
1961	–	–	Mild generalized lymphatic hyperplasia in several organs (liver, heart, kidney, spleen and GALT), chronic active pneumonia
8768	–	–	Severe follicular hyperplasia of palatine tonsil and spleen, mild hyperplasia of LNs, moderate interstitial pneumonia, moderate chronic active gastroenteritis
9038	–	Lung: *Pneumocystis jirovecii*	Mild generalized hyperplasia of LNs, spleen and GALT, mild chronic active gastroenteritis, active pneumonia
1891T	–	–	Mild generalized follicular hyperplasia of LNs
1948T	–	–	Mild generalized hyperplasia of LNs and spleen, mild-to-moderate chronic active gastroenteritis

* LN, lymph node; GALT; gut-associated lymphoid tissue; BALT, bronchial-associated lymphoid tissue.

### Loss of CD4^+^ T-cells, SIV antibody profile and extent of B-cell activation distinguish controllers from non-controllers

**Fig. 2.**
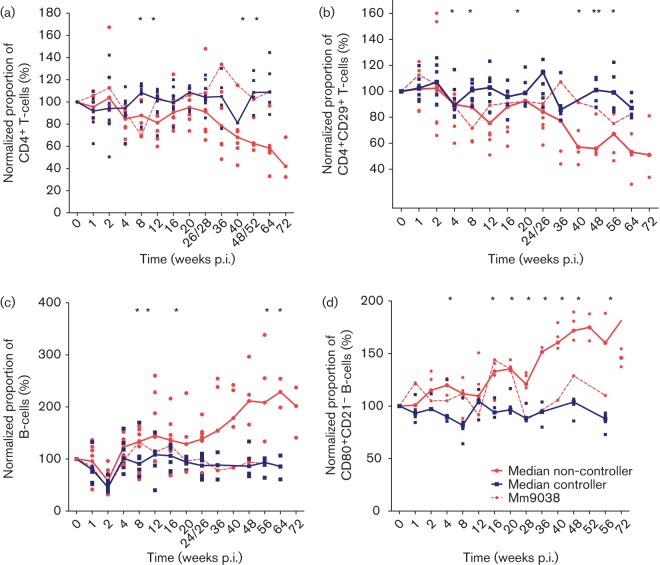
Lymphocyte subsets in SIVmac239ΔNU-infected rhesus macaques. Median values of controllers (blue) and non-controllers (red) are indicated by straight lines. Data of individual animals are shown by dots. Mm9038, which displays the phenotype of a viraemic controller, is shown by a dashed line. Asterisks mark significant differences between controllers and non-controllers (including Mm9038) calculated by the Mann–Whitney test (*P*<0.05). (a) Proportion of CD4^+^ T-cells normalized to mean of three values before infection. (b) Proportion of CD4^+^CD29^+^ T-cells normalized to mean of three values before infection. (c) Proportion of B-cells normalized to mean of three values before infection. (d) Proportion of CD80^+^CD21^–^ B-cells normalized to mean of three values before infection.

We investigated several immune parameters in order to define early immunological factors determining the disease course. First, we analysed lymphocyte subsets in blood by flow cytometry. In order to control for interindividual variation all data were related to individual pre-infection values. The percentage of CD4^+^ T-cells remained stable in all controllers. In contrast, the non-controllers showed a reduction in the percentage of CD4^+^ T-cells except for Mm9038 (Fig. 2a), which also maintained physiological levels of other immune parameters. The difference in CD4^+^ T-cell frequency between controllers and non-controllers became significant early after infection (8 and 12 weeks p.i.; *P*<0.05, Mann–Whitney test). Likewise, absolute CD4^+^ cell counts were reduced in the animals with detectable viral replication and dropped below 200 cells µl^−1^ in Mm1948 and Mm1961 (data not shown). Similarly, the proportion of CD29^high^-expressing memory CD4^+^ T-cells, an early marker for disease progression (Blatt *et al.*, 1995; Dolan *et al.*, 1995; Kneitz *et al.*, 1993), was decreased in monkeys with overt viral replication (Fig. 2b).

Interestingly, the strongest alterations after SIVmac239ΔNU infection were observed within the B-cell population. Whilst the proportion of B-cells in blood dropped in all animals at 2 weeks p.i. (Fig. 2c), a differential increase was observed during the following weeks. Controllers regained pre-infection levels 2 weeks later and maintained the levels, whereas in the non-controllers this population expanded continuously. The difference between the two groups reached significance at 8 weeks p.i. (*P*<0.05, Mann–Whitney test). Increased activation of B-cells was further investigated by a phenotypic analysis of these cells. Macaque B-cells can be subdivided into two mutually exclusive subsets according to the expression of CD21 and the co-stimulatory molecules CD80 or CD86 (Sopper *et al.*, 1997). CD21^high^ cells represent terminally differentiated cell (resting cells), whereas CD21^low^ cells represent activated (differentiated) B-cells (Niino *et al.*, 2009). In a subset of nine macaques, we determined levels of CD80^+^CD21^−^ B-cells. Activated CD80^+^CD21^−^ cells increased in non-controllers, whereas in the controllers these cells remained stable (Fig. 2d). The difference between controllers and non-controllers became significant by 16 weeks p.i. (*P*<0.04, Mann–Whitney *U* test). These findings indicate a strong activation of B-cells in the non-controllers.

**Fig. 3.**
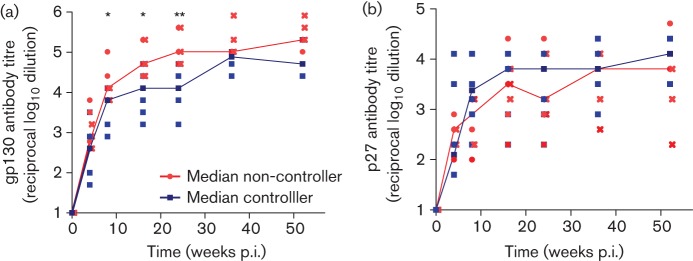
SIV-specific serum antibody titres after infection with SIVmacΔNU: individual titres (reciprocal log_10_ dilution) against (a) SIVgp130 and (b) SIVp27 at the indicated time points. Controllers are shown in blue, non-controllers in red. gp130 titres differ between controllers and non-controllers significantly at 8, 16 and 24–26 weeks p.i., and are marked by asterisks (*P*<0.03, Mann–Whitney *t*-test). Titres from three macaques with the highest viral load are indicated by crosses.

SIV-binding antibody titres were also determined (Fig. 3). Early in infection (8 to 24/26 weeks p.i.), non-controllers had significantly higher Env (gp130)-binding antibody titres compared with controllers (*P*<0.05, Mann–Whitney test; Fig. 3a). In contrast, the kinetics of Gag (p27)-specific antibody titres were similar for both groups (Fig. 3b). Only late after infection did Gag antibodies titres start to decline in those animals with the highest viral load (Fig. 3a), consistent with the known prognostic value of low-Gag-binding antibodies (Putkonen *et al.*, 1992; Weber *et al.*, 1987).

### No evidence for increased viral virulence after transfusion of blood from monkeys with progressing disease

Previous studies have shown that partial repair of defective *nef* genes is associated with increased viral load in some HIV-infected patients or SIV-infected macaques (Carl *et al.*, 2000; Chakrabarti *et al.*, 2003; Sawai *et al.*, 2000; Whatmore *et al.*, 1995). To investigate whether the *nef* deletion was still intact, *nef* PCR and direct DNA sequencing of the PCR products were carried out in the two macaques with the highest viral load, Mm1891 and Mm1948. We found no evidence of refilling of *nef* sequences, and the DNA sequences around the *nef* deletion site were identical between the infecting virus and those analysed in peripheral blood mononuclear cells (PBMCs) of the macaques progressing to disease (data not shown).

To investigate whether the virus had changed its virulence in monkeys with progressing disease, a transfusion experiment was performed. Aliquots of 5 ml of peripheral blood of Mm1891 or Mm1948 spiked with 10^7^ autologous lymph node cells were transfused into two naive animals (Mm1891T and Mm1948T) at 54 weeks after donor infection. In order to avoid potential effects attributable to escape mutants that may have developed in the donor macaques, recipient animals carried MHC genotypes different from the donors and lacked MHC class I genes known to be associated with superior viral control such as Mamu-A1*01 or -B*17 (Table S1). However, one monkey (Mm1948T) carried MHC class II *DRB* alleles that have been reported to be over-represented in elite controllers (Giraldo-Vela *et al.*, 2008).

**Fig. 4.**
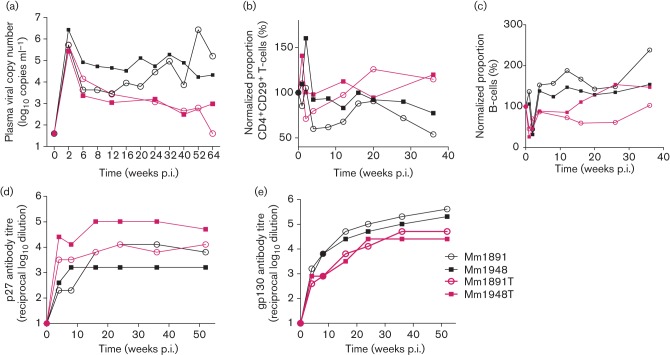
Comparison of virological and immunological data of transfused macaques. (a) Plasma viral RNA copies (log_10_), (b) normalized percentage of CD4^+^CD29^+^ T-cells, (c) normalized percentage of B-cells, and (d) SIVp27 and (e) SIVpg130 antibody titres (reciprocal log_10_ dilution obtained by ELISA) in donor monkeys (Mm1891, Mm1948) and transfused macaques (1891T, 1948T).

After infection, viral RNA copy numbers declined steadily to undetectable levels in Mm1891T and at least by three orders of magnitude in Mm1948T during the observation period (Fig. 4a). The cell-associated viral load declined with time, and reached the detection level of the assay at 36 and 44 weeks p.i. (data not shown). Similar to the controllers, the percentage of CD4^+^CD29^+^ cells remained stable during the observation period (Fig. 4b). Likewise, the portion of B-cells did not change conspicuously, albeit an increase was later seen in infection in Mm1948T (Fig. 4c). Furthermore, in contrast to the donor monkeys, the transfused macaques developed high Gag and low Env antibody titres (Fig. 4d, e). In contrast to the donor macaques, which developed severe lymphadenopathy and other signs of AIDS-related symptoms, the two transfused animals displayed only mild symptoms at euthanasia (58 and 61 weeks p.i.) (Table 2). Considering the high degree of immune activation probably induced by the inoculation of foreign antigen, the transferred virus still retained the attenuated phenotype of the original clone.

### MHC genotypes do not exclusively explain the differential disease progression

Genes encoded in the MHC class I region are important genetic determinants of disease progression in pathogenic SIV infection. Previously, we have identified MHC class I haplotypes and single MHC class I sequences associated with rapid, moderate and slow disease progression (Sauermann *et al.*, 2008). MHC haplotypes were known for the nine macaques originating from the German Primate Center (DPZ) colony. The remaining animals from other breeding facilities were typed in detail by employing 49 MHC primer pairs specific for single MHC alleles or allelic lineages.

**Fig. 5.**
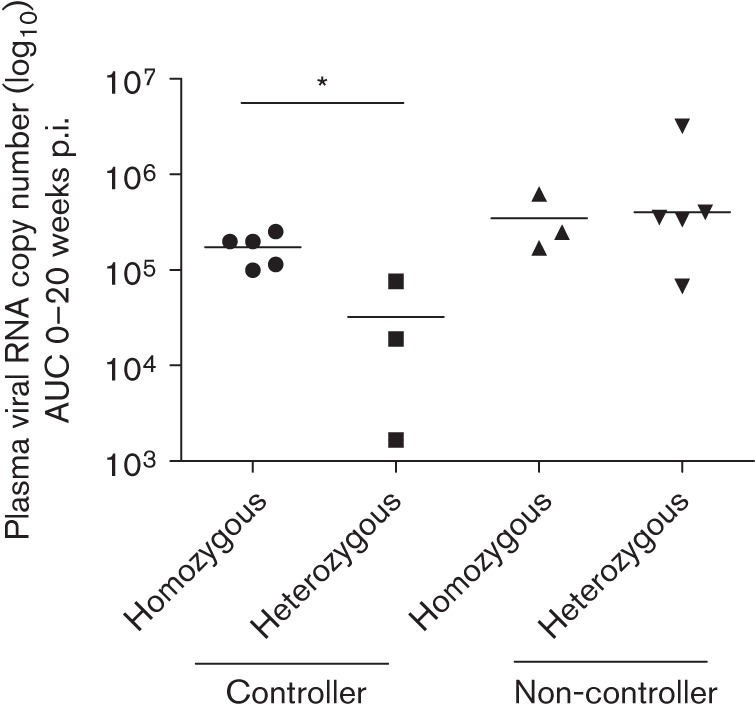
Plasma RNA copy number from 0 to 20 weeks p.i. depicted as area under curve (AUC) stratified against MHC class II *DQB1-DRB* haplotype homo- or heterozygosity in controllers and non-controllers. MHC *DQB1-DRB* homozygous controllers had a significantly higher viral load compared with heterozygous controllers (*P* = 0.036, Mann–Whitney test). Geometric means are shown by a horizontal line.

Four of eight controllers, but none of the non-controllers, carried MHC class I alleles reported to be associated with slow or moderate disease progression (*P* = 0.02, χ^2^ test; Tables 1 and S1). Two controllers carried genotypes previously associated with rapid disease progression in pathogenic SIV infection. Furthermore, these two animals had MHC-identical siblings that had progressed rapidly to AIDS-like disease after infection with pathogenic SIVmac (Sauermann *et al.*, 2008). In contrast, only one out of nine non-controllers carried MHC alleles associated with rapid disease progression. The remaining animals in both groups carried MHC class I sequences with hitherto undetermined association with disease progression after SIVmac infection, such as Mamu-B*43, -B*26, -B*27 or -A1*002 (Sauermann *et al.*, 2008). The analysis of MHC class II *DQB1* and *DRB* genes revealed five *DQB1-DRB* homozygous macaques that were able to control viral replication. In contrast, only three non-controllers were homozygous for their MHC *DQB1-DRB* genotype. Homozygous macaques express only one *DQB1* gene and one or two different *DRB* genes, and thus present presumably a smaller repertoire of peptides as compared with heterozygous macaques. Interestingly, within the group of controllers, MHC class II homozygous macaques initially had a significantly higher viral load as compared with MHC class II heterozygous macaques (area under curve 0–20 weeks p.i.: *P* = 0.036, Mann–Whitney test, Fig. 5). The two Mamu-A1*001-positive macaques were equally distributed between the *DQB1-DRB* homozygotes and heterozygotes. This effect was not observed in macaques with median to high viral load.

### *TLR7* polymorphisms are not significantly associated with viral replication

A *TLR7* variant has been found to be associated with a more severe clinical disease course in HIV-infected human patients (Oh *et al.*, 2009). In SIV-infected rhesus macaques, we previously identified a *TLR7* genotype (c.−17C, c.13G) associated with prolonged survival and lower set-point viral load compared with the other investigated *TLR7* genotypes (Siddiqui *et al.*, 2011). Six of eight macaques controlling viral replication carried the genotype c.−17C, c.13G associated with prolonged survival (75 %) and four of nine (55 %) with moderate to high viral load, including the two with the highest viral load. Thus, although the *TLR7* genotype associated with slower disease progression is under-represented in macaques with high viral load, this does not explain the non-controller phenotype.

### Genotypes associated with protection after challenge with SIVmac251

Twelve macaques were challenged with SIVmac251 (Freissmuth *et al.*, 2010; Tenner-Racz *et al.*, 2004). Five macaques did not replicate SIVmac251 significantly at the time of necropsy and were regarded as protected (Mm1937, Mm1964, Mm1982, Mm8637 and Mm8785) (Freissmuth *et al.*, 2010). Four controllers were protected and carried divergent MHC class I genotypes, but were – in contrast to the four unprotected controllers – homozygous for MHC class II genotypes and carried a *TLR7* genotype associated with slow disease progression (Fisher’s exact test: *P* = 0.028, *P* = 0.015 all challenged macaques). The protected controllers showed a trend to replicate SIVmacΔ*nef* (2–6 weeks p.i.) more vigorously as compared with the unprotected controllers (*P* = 0.057, area under curve, Mann–Whitney test). One (Mm8637) of three investigated non-controller macaques did not replicate SIVmac251 at necropsy. Among the non-controllers, Mm8637 had the lowest peak viraemia and the lowest cell-associated virus load in lymph nodes shortly before challenge (Table S2). Among the controllers, cell-associated viral load in lymph nodes before challenge (Table S2) did not correlate with protection. Moreover, cell-associated viral load in lymph nodes was generally higher compared with PBMCs (see also Berry *et al.*, 2011; Fukazawa *et al.*, 2012).

## Discussion

The protective immune responses induced by infection with *nef*-deleted SIVs have been considered to be instructive for the development of an effective AIDS vaccine (Daniel *et al.*, 1992; Koff *et al.*, 2006; Whitney & Ruprecht, 2004). This appears to be of particular importance, as clear correlates of protection against HIV infection are only beginning to emerge (de Souza *et al.*, 2012; Haynes *et al.*, 2012). Interestingly, protection conferred by live attenuated viruses can be obtained in the presence of low or even absent adaptive immune responses (Mansfield *et al.*, 2008; Stebbings *et al.*, 2005; Wade-Evans *et al.*, 2001). Recent reports have shown that antibody-dependent cell-mediated cytotoxicity and CD4^+^/CD8^+^ T-cell responses, especially in lymph nodes, are responsible for conferring protection against challenge with pathogenic SIV (Alpert *et al.*, 2012; Fukazawa *et al.*, 2012). An obstacle to using *nef*-deleted viruses for AIDS vaccine research in non-human primates is their varying replication pattern, especially in rhesus macaques of Indian origin, affecting vaccine efficacy, e.g. by fostering the generation of recombinant viruses (Gundlach *et al.*, 2000; Reynolds *et al.*, 2008), or by themselves causing AIDS-like disease in non-controllers (Hofmann-Lehmann *et al.*, 2003).

In this study, our aim was to analyse whether immunological, host genetic factors or gross changes in the viral genome were associated with differential viral load and disease progression in rhesus macaques infected with SIVmac239ΔNU. Plasma viral RNA copy numbers were reduced initially in all macaques, but viral loads at set point finally showed considerable differences, with mostly non-detectable levels in controllers and plasma viral load >10^4^ copies ml^−1^ in non-controllers. Controllers had stable CD4^+^ and B-cell counts, and did not display symptoms of immunodeficiency during the observation period. The non-controllers replicated SIVmac239ΔNU at moderate to high levels and the three with the highest plasma viral load had declining CD4^+^ T-cells. They were euthanized with signs of immunodeficiency, which can be related directly to the SIVmacΔ*nef* infection. Plasma RNA copy numbers in controllers and non-controllers already differed significantly at peak viraemia. As at this point in time adaptive immune responses play only a minor role in controlling viral replication, the intrinsic capability to replicate the virus may have influenced the outcome of infection.

Non-controllers already had increasing numbers of activated B-cells in the early stages of infection, elevated SIV Env as well as lower SIV Gag antibody titres. Thus, B-cell responses deteriorated in the non-controllers. B-cell dysfunction represents a hallmark of HIV and SIV infection (De Milito, 2004; Moir & Fauci, 2009). Some effects of B-cell dysfunction, such as hyperactivation and impairment of immunoglobulin class switching, have been attributed directly to Nef (Moir & Fauci, 2010; Qiao *et al.*, 2006; Swingler *et al.*, 2008; Xu *et al.*, 2009). As the macaques had been infected with a *nef*-deleted virus, B-cell hyperactivation seen in these monkeys could not have been caused by Nef, but through a direct consequence of viral replication.

Our transfusion experiment has shown that the virus from immunocompromised monkeys retained an attenuated phenotype after passage in susceptible animals. Sequence analyses revealed that the virus had kept the *nef* deletion. Viral loads in transfused monkeys were reduced by at least 20-fold compared with those in the donor animals in the chronic phase of infection. No decrease of CD4^+^CD29^+^ T-cells was observed and the proportion of B-cells remained stable. Furthermore, both monkeys had rather low Env and Gag antibody titres similar to controllers. These findings contrast with other reports demonstrating that *in vivo* passage increases the pathogenicity of SIVmac or SHIV (simian-human immunodeficiency virus) (Holterman *et al.*, 1999; Tan *et al.*, 1999), or showing that the genome of viruses with impaired or deleted Nef function can acquire compensatory mutations that increase pathogenicity (Alexander *et al.*, 2003). A difference from the other reports is, however, that the transfused macaques of this study received material from monkeys that had not developed AIDS-like symptoms. Although the transfused macaques did not show all features of perfect controllers, viral replication was clearly attenuated compared with the donor macaques. Therefore, we conclude that host factors played the major role in determining the extent of viral replication in this study.

We did not find a common strong genetic predictor of disease progression in Δ*nef*-infected animals. Although MHC class I alleles associated with some control of SIVmac infection (Sauermann *et al.*, 2008) were found exclusively among the controllers, two carried MHC class I genotypes associated with rapid disease progression in SIVmac-infected macaques. Similarly, genotyping of *TLR7* variants described to be correlated with disease progression in pathogenic SIV infection (Siddiqui *et al.*, 2011) did not provide clear associations in our study with *nef-*deleted virus.

Results only became significant when genetic and viral load data were related to protection from infection with SIVmac251. Four of five animals (Freissmuth *et al.*, 2010) that did not strongly replicate the challenge virus SIVmac251 at necropsy were – in contrast to the other four controllers and the majority of non-controllers – homozygous for MHC class II and carried a *TLR7* genotype associated with slow disease progression, but had divergent MHC class I genotypes. Thus, in accordance with other reports, cytotoxic T lymphocyte (CTL) responses may play only a minor role in protective immune responses after SIVmacΔ*nef* infection (Mansfield *et al.*, 2008; Stahl-Hennig *et al.*, 2007; Stebbings *et al.*, 2005). The fifth protected animal was a non-controller (Mm8637). The slight over-representation of the TLR7 polymorphism in protected macaques may be related to differential immune activation. The association of Mhc class II genotypes with protection may be linked to the fact that early viral replication in controllers was strongest in macaques homozygous at the MHC class II *DQB-DRB* region. The effect was probably masked in the non-controllers by the vigorous viral replication. This result matches in part observations that the extent of viral replication and/or the degree of attenuation determine protection against pathogenic SIVmac (Koff *et al.*, 2006; Whitney & Ruprecht, 2004).

Among the non-controllers, only the macaque with the lowest peak viraemia was protected from infection with SIVmac251; others progressed to early signs of immune dysfunction or replicated finally SIVmac251. Moreover, in contrast to Fukazawa *et al.* (2012), we found no evidence that increased presence of SIV in lymph nodes before challenge was associated with protection. Our results suggest that a certain degree of early viral replication followed by an efficient immune response that most likely includes T-cell responses in lymph nodes was associated with protection. Further analyses will be required to investigate whether homozygosity for MHC class II genes or polymorphisms in genes linked to this region influence initial viral replication and protection. Moreover, viral replication may be determined by host factors which differ from those after pathogenic SIV infection. In the context of an infection with Nef-impaired or *nef*-deleted virus, the impact of some host factors may be altered because of the slower replication kinetics of the virus or because they are not inactivated by Nef, such as tetherin and cell-surface receptors, e.g. CCR5 or CXCR4 (Hrecka *et al.*, 2005; Landi *et al.*, 2011; Michel *et al.*, 2006; Zhang *et al.*, 2009). Identification of these host factors will provide further insights into the mechanisms associated with viral control.

## Methods

### 

#### Rhesus monkeys.

Nineteen rhesus macaques (*M. mulatta*) of Indian origin, seronegative for SIV, simian T-cell leukemia virus type 1 and D-type virus, were infected with SIVmac239ΔNU (Gundlach *et al.*, 1997). Eleven animals were inoculated intravenously with 300 TCID_50_ SIVmac239ΔNU. Six macaques were infected by atraumatic application of 10^5^ TCID_50_ SIVmac239ΔNU onto the tonsils (Stahl-Hennig *et al.*, 2007; Tenner-Racz *et al.*, 2004). Another two monkeys were transfused with 5 ml citrated blood spiked with 10^7^ lymph node cells from two donor monkeys 54 weeks p.i. with SIVmac239ΔNU. The overall number of transferred cell-associated infectious units was 25 600 for 1891T and 21 440 for 1948T. The housing and treatment protocols of the DPZ follow the German Animal Welfare Act strictly, which in turn complies with European Union guidelines on the use of non-human primates for biomedical research. An external ethics board empowered by the Lower Saxony State Office for Consumer Protection and Food Safety approved all experiments under the project licences 509.42502/08-02.95 and 509.42502/08-13.98. Blood samples were drawn from monkeys which were anaesthetized intramuscularly with 10 mg ketamine (kg body weight)^–1^. Twelve macaques were later inoculated with SIVmac251 as described previously (Freissmuth *et al.*, 2010; Tenner-Racz *et al.*, 2004).

#### Cell-associated viral load and plasma viral RNA copy number determination.

The cell-associated virus load in peripheral blood and lymph nodes was determined by a limiting-dilution co-cultivation assay as described previously (Stahl-Hennig *et al.*, 1996; Stahl-Hennig *et al.*, 1992). Viral RNA copies in plasma samples by were determined by competitive quantitative RNA-PCR (Ten Haaft *et al.*, 1998).

#### Lymphocyte phenotyping and counting.

Three-colour flow cytometry was performed as described previously (Sopper *et al.*, 2000). Briefly, 50 µl citrated blood was incubated with the antibodies at pre-titrated concentrations for 30 min at 4 °C. The following antibody combinations were used together with the anti-monkey CD3 antibody FN18 (biotin conjugated using standard techniques; M. Jonker, TNO, Rijswijk, The Netherlands) to determine T-cell subsets: CD29 (4b4, FITC conjugated; Coulter) and CD4 [OKT4, phycoerythrin (PE) conjugated; Ortho); CD8 (RPAT8, FITC coupled; Pharmingen) and CD69 (L78, PE conjugated; Becton Dickinson); CD8–FITC and Ki-67 (PE conjugated, Dako). For phenotypic characterization of B-cells (CD20, 2H7; Cy-Chrome conjugated; Pharmingen) antibodies directed against CD21 (B-IY4, FITC conjugated; DPC) and CD80 (L307.4, PE coupled; Becton Dickinson) were used, which define different subsets (Sopper *et al.*, 1997). After lysing of erythrocytes and fixation of the cells with FACS lysing solution (Becton Dickinson), bound biotinylated antibodies were detected with streptavidin-coupled Cy-Chrome (Pharmingen). For intracellular staining with Ki-67, cells were fixed using 3 % formaldehyde and membranes were permeabilized using 0.3 % Triton. Cells were analysed on a FACScan flow cytometer (Becton Dickinson) using Lysis II and CellQuest software. Quadrants were set according to the staining pattern obtained with isotype-matched control antibodies, except for the bimodal distribution of CD29 where markers were set between the two CD29^low^- and CD29^high^-expressing populations. For the determination of absolute numbers of lymphocyte subsets, the percentage of the respective population within a forward and side light scatter gate including CD3^+^ T-cells, CD20^+^ B-cells and CD3^+^CD8^+^ NK cells (total lymphocytes) was calculated. This proportion was combined with the absolute number of lymphocytes obtained using a Coulter counter.

#### SIV-binding antibodies.

To measure humoral SIV-specific responses, a standard ELISA for the detection of antibodies against the SIV polypeptides gp130 SU and p27 capsid antigen (Stolte-Leeb *et al.*, 2006) in a limiting-dilution format was performed. For antigen coating, recombinant monomeric SIVgp130 (EVA670) and SIVp27 (EVA643) kindly provided by the Centre for AIDS Reagents, National Institute for Biological Standards and Control (NIBSC), UK, were used. ELISA titres refer to the serum dilution yielding a twofold higher absorbance compared with that obtained with sera collected before infection.

#### Analyses of viral *nef* sequences.

To investigate whether the *nef* deletions were still present in the animals with a high viral load (Mm1948 and Mm1891), DNA was extracted from lymphocytes obtained at 64 and 88 weeks p.i. *nef* PCR was performed as described previously (Gundlach *et al.*, 1997). Size analysis was performed by agarose gel electrophoresis and DNA sequence analysis of the purified PCR products was performed by SeqLab.

#### Genotyping.

Typing of the *DQB1* alleles was performed by PCR-RFLP or by DNA sequence analysis of exon 2 as described previously (Vigon & Sauermann, 2002). For *DRB* typing, PCR products of exon 2 were separated by denaturing gradient gel electrophoresis, followed by DNA sequence determination of the eluted and reamplified PCR products (Khazand *et al.*, 1999). MHC class I typing with 49 MHC class I typing primers was performed essentially as described previously (Sauermann *et al.*, 2008).

Typing for *TLR7* single nucleotide polymorphisms c.−17C>T and c.13G>A was performed as described previously (Siddiqui *et al.*, 2011).

#### Statistical analysis.

GraphPad Prism version 5 was used for statistical analysis. The Mann–Whitney *U* test was applied for comparisons between groups. Correlations were calculated using Spearman rank correlation. The statistical tests employed are indicated along with each *P* value.
